# Hyperkalemia: pathophysiology, risk factors and consequences

**DOI:** 10.1093/ndt/gfz206

**Published:** 2019-12-04

**Authors:** Robert W Hunter, Matthew A Bailey

**Affiliations:** British Heart Foundation Centre for Cardiovascular Science, University of Edinburgh, Queen’s Medical Research Institute, Edinburgh BioQuarter, Edinburgh, UK

**Keywords:** aldosterone, arrhythmia, hyperkalemia, potassium, renin–angiotensin

## Abstract

There have been significant recent advances in our understanding of the mechanisms that maintain potassium homoeostasis and the clinical consequences of hyperkalemia. In this article we discuss these advances within a concise review of the pathophysiology, risk factors and consequences of hyperkalemia. We highlight aspects that are of particular relevance for clinical practice. Hyperkalemia occurs when renal potassium excretion is limited by reductions in glomerular filtration rate, tubular flow, distal sodium delivery or the expression of aldosterone-sensitive ion transporters in the distal nephron. Accordingly, the major risk factors for hyperkalemia are renal failure, diabetes mellitus, adrenal disease and the use of angiotensin-converting enzyme inhibitors, angiotensin receptor blockers or potassium-sparing diuretics. Hyperkalemia is associated with an increased risk of death, and this is only in part explicable by hyperkalemia-induced cardiac arrhythmia. In addition to its well-established effects on cardiac excitability, hyperkalemia could also contribute to peripheral neuropathy and cause renal tubular acidosis. Hyperkalemia—or the fear of hyperkalemia—contributes to the underprescription of potentially beneficial medications, particularly in heart failure. The newer potassium binders could play a role in attempts to minimize reduced prescribing of renin–angiotensin inhibitors and mineraolocorticoid antagonists in this context.

## INTRODUCTION

The introduction of new potassium binders (patiromer and zirconium cyclosilicate) has refocused attention on hyperkalemia. There have also been significant recent advances in our understanding of the mechanisms that maintain potassium homoeostasis and the clinical consequences of hyperkalemia. In this article we aim to present these recent advances in the context of a concise review of the pathophysiology, risk factors and consequences of hyperkalemia. We will highlight aspects that are of particular relevance for clinical practice.

## PATHOPHYSIOLOGY OF HYPERKALEMIA

### Principles of potassium homoeostasis: a battle waged on two fronts

#### The three-pronged response to an acute potassium load

The extracellular potassium concentration, [K^+^]_e_, is kept under tight control to maintain the resting membrane potential (RP) of excitable cells. This control is under continual threat from two sources of potassium influx. The first is internal: 98% of total body potassium (3–4 mol) is stored within cells, predominantly skeletal muscle. The second is external: our potassium-rich diet. A modern Western diet contains ∼120 mmol potassium per day and throughout most of our evolutionary history this was a great deal more (∼300 mmol per day in the palaeolithic diet) [1].

Consequently we have evolved robust mechanisms to defend against potassium influx into the extracellular space (reviewed in McDonough and Youn [2]). Without this defence, we would rarely make it past breakfast: a banana smoothie delivering 35 mmol of potassium to an extracellular fluid volume of 12 L would induce a potentially fatal [K^+^]_e_ increase of ∼3 mM. We survive the banana smoothie because of a rapid response that shifts potassium into cells and into the urine. Thus the large intracellular potassium store constitutes a potential threat but is also a lifesaving buffer.

Recent studies in healthy human subjects have helped to delineate three prongs to the response to a dietary potassium load [3]. When subjects were administered 35 mmol K^+^, plasma [K^+^] rose by ∼0.5 mM and was accompanied by increases in plasma [aldosterone] and renal K^+^ excretion (Prong 1: the classic aldosterone-dependent negative feedback loop). When the same potassium load was administered as part of a complex meal, there was no change in plasma [K^+^], probably reflecting insulin-mediated transcellular potassium shifts (Prong 2). Despite there being no change in venous plasma [K^+^], there was an increase in renal potassium excretion, which was not prevented by treatment with the mineraolocorticoid receptor (MR) antagonist eplerenone. This provides evidence for an aldosterone-independent, gut-to-kidney feedforward kaliuretic signal (Prong 3).

Although only recently confirmed in humans, this mode of feedforward control has been known about for several decades in sheep [4] and has been studied in some detail in rodents [5, 6]. In one particularly meticulous study, potassium loads were administered to rats *via* an enteral or intravenous route in such a way as to induce identical increases in plasma [K^+^]. Enteral loads elicited a kaliuretic response of greater magnitude [6]. The gut-responsive ‘kaliuretic factor’ has not been identified. It has been hypothesized to be a peptide hormone or a centrally mediated reflex [7], but one cannot discount the possibility that there is no mystery factor and instead the error signal driving kaliuresis is a small increase in the potassium concentration in the renal peritubular capillaries, not readily detectable by venous sampling. Testing a panel of known gut or pituitary peptide hormones did not reveal a likely culprit [6].

Whatever the mechanism(s), the clinical ramifications of these physiological observations have not been explored fully. Is hyperkalemia more likely to be provoked by intravenous than by oral potassium supplements? Could manipulation of diet *composition*, rather than mere potassium content *per se* prevent hyperkalemia in patients with end-stage renal disease? If we could determine the molecular basis of the gut potassium ‘sensor’, then could we target this with novel drug therapies?

#### Chronic potassium homoeostasis: not just aldosterone

Plasma [K^+^] is controlled by aldosterone in a negative feedback loop. Aldosterone is synthesized by aldosterone synthase (AS) in the adrenal cortex in response to high [K^+^]_e_ and angiotensin II. It acts in the distal nephron to increase the activity of sodium (Na)–K–adenosine triphosphatase (ATPase) pumps and epithelial sodium channel (ENaC), renal outer medullary potassium (ROMK) and large (‘big’) potassium (BK) channels to promote kaliuresis [8]. (We discuss the molecular basis of renal potassium excretion in more detail below.)

Aldosterone is the dominant factor regulating plasma [K^+^], but it is not the only one. Two mouse models have been used to explore the extent to which aldosterone is necessary for potassium homoeostasis: AS-null mice (which are unable to synthesize aldosterone) and kidney-specific MR-null mice (which possess kidneys that are unable to respond to aldosterone signalling) [9, 10]. Both models develop hyperkalemia when challenged with supraphysiological potassium loads. However, AS-null mice can maintain a normal plasma [K^+^] in the face of physiological (2%) dietary K^+^, demonstrating that aldosterone-independent pathways can stimulate kaliuresis in this context.

Chronic potassium homoeostasis is maintained not only by fine-tuning renal K^+^ excretion, but also by modulating transcellular potassium shifts. The magnitude of (net) transcellular potassium shifts can be measured experimentally using a ‘potassium clamp’, in which the rate that potassium exits the vascular space is inferred from the rate of potassium infusion required to clamp plasma [K^+^] at a constant level. This approach was used in the rat to demonstrate key features of the insulin–potassium homoeostatic system [11]. After short-term potassium depletion, insulin-induced potassium shifts were markedly reduced (without any change in insulin-mediated glucose clearance). Thus the gain of this system is modified by potassium status and is regulated independently from insulin–glucose homoeostasis.

#### It’s complicated!

Of course, the above model is an over-simplification. Potassium homoeostasis is not independent from the many other facets of systemic physiology and we are continually learning about new pieces in the puzzle. One particularly intriguing story that has emerged in recent years is that of the circadian influences on potassium excretion. Renal potassium excretion follows a circadian rhythm, being highest around noon and lowest around midnight. Renal tubular cells possess an intrinsic molecular clock that is now well-characterized. This is synchronized with the central (brain) clock, in part through glucocorticoid signalling [12].

It follows that the risk of hyperkalemia is almost certainly influenced by the *timing* of meals, potassium loads and drug administrations. Could this be exploited to minimize the risk of hyperkalemia in high-risk patients?

### Hyperkalemia from transcellular potassium shifts

The huge size of the intracellular potassium store means that transcellular shifts can have large and rapid effects on plasma [K^+^]. Potassium shifted from the intra- to the extracellular space are induced by acute metabolic acidosis and opposed by insulin and β-adrenergic signalling [13]. Widespread cell death (as in tumour lysis or rhabdomyolysis) may also release potassium from the intracellular space.

Transcellular shifts can be quantitatively more important than external potassium load, as was demonstrated by randomized controlled trials (RCTs) of perioperative intravenous fluid therapy in kidney transplant recipients. Patients randomized to receive 0.9% sodium chloride (NaCl; containing no potassium) had a greater incidence of hyperkalemia than those randomized to receive plasmalyte-148 (containing 4 mM potassium) [14, 15]. The likely explanation for this apparent paradox is that chloride-rich 0.9% NaCl induces metabolic acidosis, whereas buffered plasmalyte-148 does not.

### Hyperkalemia from defective potassium excretion in the distal nephron

Ninety percent of excreted potassium exits via the kidneys and the kidneys have a remarkable capacity to increase potassium excretion in the face of potassium excess [16]. Consequently hyperkalemia is almost never encountered clinically in the context of normal renal function and a normal adrenal–kidney axis.

Physiological control of potassium excretion is exercised in the aldosterone-sensitive distal nephron (reviewed in McDonough and Youn [2] and Welling [8]). An understanding of the molecular pathways of potassium excretion can help in understanding the clinical insults that induce hyperkalemia. Potassium is secreted through renal tubular cells via the sodium–potassium pump in the basolateral membrane and at least four different types of ion channels in the apical membrane. The most well-studied of these are the ROMK channel and the BK channel.

#### ROMK channels and the coupling of sodium and potassium transport in the distal nephron

ROMK channels are expressed in principal cells alongside the ENaC. This arrangement means that potassium excretion in the distal nephron is coupled to sodium reabsorption. Na^+^ reabsorption through the ENaC generates a lumen-negative potential, favouring K^+^ excretion. When sodium influx through the ENaC is high (e.g. in response to aldosterone signalling), potassium efflux is also high. Conversely, when sodium influx is low (as in volume depletion, when sodium delivery to the distal nephron is limited), potassium efflux is diminished. In potassium excess, hyperkalemia is avoided because ROMK is upregulated through aldosterone-dependent and -independent pathways [8].

Sodium delivery to the distal nephron is therefore an important determinant of potassium excretion. Except in extreme volume depletion, tubuloglomerular feedback ensures that a constant load of NaCl is delivered to the post-macular renal tubule [17]. Therefore the supply of sodium to the ENaC in the connecting tubule and collecting ducts is determined primarily by the activity of the distal electroneutral sodium reabsorption pathways: sodium chloride cotransporter (NCC) in the distal convoluted tubule and the sodium-dependent Cl^−^/HCO_3_^−^ exchanger (NDCBE) in the β-intercalated cell, functionally coupled with pendrin [18]. Reciprocal studies in mice show the importance for potassium homoeostasis of coordinated crosstalk between these distal sodium transporters: double genetic deletion of NCC and NDCBE caused hypokalemia in mice, which could not be explained solely on the basis of activation of the renin–angiotensin–aldosterone system [19]: the expression of ENaC, ROMK and BK proteins was increased as was the natriuretic effect of amiloride. Conversely, transgenic mice carrying the Q562E mutation in WNK4 that causes pseudohypoaldosteronism type II (PHAII) mutation were hyperkalemic due to combined activation of NCC and pendrin/NDCBE and ‘reduced’ potassium secretion by the principal cell [20].

The balance between electroneutral and electrogenic distal Na^+^ reabsorption determines net potassium excretion. This physiological switch is manipulated by the mechanisms that maintain potassium homoeostasis. Potassium channels (Kir4.1) in the basolateral membranes of distal convoluted tubule cells act as ‘potassium sensors’, activating NCC in response to potassium depletion [21, 22]. Furthermore, NCC activity can be controlled by phosphorylation/dephosphorylation of critical residues in the N-terminus. Dephosphorylation, suppressing NCC activity, helps to enhance potassium excretion after a potassium-rich meal: the effector arm of the gut–kidney feedforward loop [2]. Dephosphorylation is also observed following acute hyperkalemia in the rat [23], and the urinary abundance of phosphorylated NCC is negatively correlated with plasma [K^+^] in humans [24]. NCC is directly activated by aldosterone [25–27] but can also be controlled by aldosterone-independent mechanisms. For example, potassium restriction stimulates NCC activity in kidney-specific MR-null mice [10].

#### BK channels and their regulation by urinary flow rate and alkalinization

BK channels are expressed in principal and intercalated cells. These are activated by high tubular flow rates and thus mediate flow-induced potassium secretion. The ‘flow sensor’ has recently been determined: bending of the primary cilium on the principle cell opens transient receptor potential vanilloid type 4 channels, causing an influx of calcium that in turn activates BK channels [28]. BK channels are also upregulated in response to potassium loading. The mechanism here is fascinating. BK channels are formed from two subunits, both of which are required for channel activity. Aldosterone upregulates the expression of the α-subunit and bicarbonate upregulates expression of the β-subunit. Therefore it has been argued that regulation of BK channel activity is critical in the response to a ‘Palaeolithic’ low-sodium, high-potassium, alkaline diet [8]. In support of this, mice fed a low-sodium, high-potassium diet become hyperkalemic when they are also given an acid load [29].

#### Clinical implications

**FIGURE 1:**
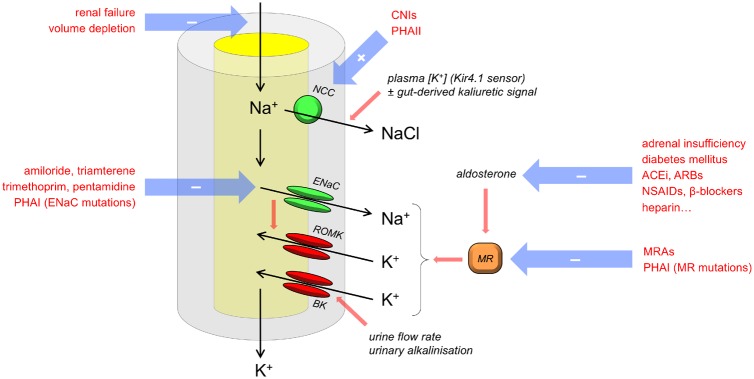
Pathogenesis of hyperkalemia: mechanisms in the distal nephron. Factors that can cause hyperkalemia are in red text. These perturb one or more of five key variables: glomerular filtration, urine flow, sodium delivery to the distal nephron, expression of aldosterone-sensitive ion channels and transporters and urinary pH. PHAI, type I pseudohypoaldosteronism; CNI, calcineurin inhibitor.

These molecular processes explain why hyperkalemia invariably occurs in response to stimuli that limit any of five key parameters (Figure 1): (i) glomerular filtration rate, (ii) tubular flow rate, (iii) sodium delivery to the distal nephron, (iv) the expression of potassium and sodium channels in the apical cell membrane and the sodium–potassium pump in the basolateral membrane (controlled by aldosterone signalling) and (v) urinary pH.

In clinical practice, the first three of these are frequently encountered together, explaining the pathogenesis of hyperkalemia in acute kidney injury (AKI). The normal physiological response to volume depletion drives upregulation of aldosterone synthesis, so that any reductions in (i)–(iii) are offset by increases in (iv), maintaining potassium homoeostasis. Hyperkalemia ensues when this response is prevented, for example, by renin–angiotensin system (RAS) inhibitors or MR blockers.

Hyperkalemia can occur when NCC activity is *stimulated*, leading to reduced reabsorption of sodium through the electrogenic pathway. The archetypal example of this is type II pseudohypoaldosteronism (Gordon’s syndrome), in which inherited mutations in the Wnk-SPAK kinase cascade or the KLHL3–CUL3 ubiquitin ligase complex that regulates it lead to the constitutive phosphorylation (and therefore activation) of NCC [30]. More commonly, this syndrome, characterized by hypertension, hyperkalemia and hyperchloremic acidosis, may be acquired by renal transplant recipients taking the calcineurin inhibitor tacrolimus. Tacrolimus indirectly activates NCC [31] by inactivating negative regulators in the KLHL3–CUL3 ubiquitin ligase complex [32]. Alternatively, hyperkalemia may occur when ENaC activity is *inhibited* by drugs (e.g. amiloride, trimethoprim and pentamidine) in adrenal disease (because of impaired aldosterone synthesis) or by heritable mutations inactivating ENaC or MR (type I pseudohypoaldosteronism).

### Hyperkalemia from defective extrarenal potassium excretion

In patients with healthy kidneys, the colon excretes 10% of ingested potassium; this route of excretion becomes relatively more important in patients with advanced renal failure. In a RCT comparing spironolactone, losartan and placebo in combination with lisinopril in patients with diabetic nephropathy, spironolactone caused greater elevations in serum [K^+^] than losartan, despite exerting similar effects on urinary potassium excretion, suggesting a significant effect of spironolactone on extrarenal potassium excretion [33].

For some anuric patients, the colon undoubtedly makes a clinically significant contribution to total potassium excretion [34]. However, colonic potassium excretion probably makes a modest contribution to systemic potassium homoeostasis in most patients with end-stage renal disease. There have been several small RCTs of mineralocorticoid antagonist therapy in oligoanuric hemodialysis patients. By and large, these have shown that mineraolocorticoid antagonists do increase the risk of hyperkalemia, but this is rarely severe enough to warrant discontinuation of therapy [35–37].

## RISK FACTORS FOR HYPERKALEMIA

### Quantitatively important risk factors

From the above, it is clear that the major risk factors for hyperkalemia are renal impairment - either AKI or advanced chronic kidney disease (CKD) - and any acquired or inherited defects in potassium excretion in the distal nephron. In CKD, hyperkalemia is typically encountered after the estimated glomerular filtration rate (eGFR) drops below 15 mL/min [38].

The risk factors for hyperkalemia were studied in a meta-analysis of data from >1.2 million individuals with CKD [39]. The risk of hyperkalemia (K > 5.5 mM) was strongly correlated with eGFR across the entire range of kidney function (from eGFR 15 to 105 mL/min). A decrease in eGFR of 15 mL/min approximately doubled the odds of hyperkalemia [39]. Albuminuria was also a risk factor, but the association was far weaker (odds ratio for hyperkalemia < 2 even in heavy albuminuria) [39]. Other risk factors for hyperkalemia included male sex; non-black race; lower body mass index; smoking, history of diabetes mellitus, coronary heart disease or stroke and use of angiotensin-converting enzyme inhibitor (ACEi), angiotensin receptor blocker (ARB) or potassium-sparing diuretics. Unsurprisingly, the use of thiazide or loop diuretics was protective.

### Drug therapies as risk factors for hyperkalemia

The medications associated with hyperkalemia are listed in Table 1.

**Table 1:** Drug causes of hyperkalemia

Mechanism	Drug
Defective aldosterone signalling	
Impaired renin production	β-Blockers and NSAIDs
Impaired renin–angiotensin signalling	Aliskiren, ACEis and ARBs
Impaired aldosterone synthesis	Heparin [40] and ketoconazole
MR blockade	Spironolactone and eplerenone

Defective distal electrogenic sodium reabsorption	
ENaC blockade	Amiloride, triamterene, trimethoprim [41–43], pentamidine and lithium
NCC activation	CNIs [31]

Cellular K^+^ translocation	
Changes in transcellular transporters	α-Agonists, β-blockers, digoxin, succinylcholine, isofluorane, minoxidil and somatostatin
Solvent drag in osmotic shifts	Mannitol

Exogenous potassium load	
High-potassium content	Penicillins (intravenous) [40]

CNI, calcineurin inhibitor; NSAIDs, non-steroidal anti-inflammatory drugs.

#### RAS blockade

For most patients, RAS blockade with a single agent confers a low risk of hyperkalemia. Furthermore, patients at risk of hyperkalemia can be identified by the presence of the classical risk factors discussed above. A risk score, combining information from six risk factors (male sex, baseline [K^+^], eGFR, diabetes, heart failure and use of potassium-sparing diuretics) performed well at predicting hyperkalemia after initiation of RAS inhibitors (RASis) in Swedish and American populations [44].

However, there are significant risks of hyperkalemia when the RAS is targeted with multiple agents. The risks of dual blockade were revealed by large RCTs: Aliskiren Trial in Type 2 Diabetes Using Cardiorenal Endpoints (ALTITUDE), Veterans Affairs Nephropathy in Diabetes (VA NEPHRON-D) and Ongoing Telmisartan Alone and in Combination with Ramipril Global Endpoint Trial (ONTARGET) [45–47]. The first two trials, both conducted in patients with type 2 diabetes, were terminated prematurely because of safety concerns. Hyperkalemia (>6 mM) was significantly more common in the dual-blockade group than in a single-blockade control group (11.2% versus 7.2% in ALTITUDE and 9.9% versus 4.4% in VA NEPHRON-D). This effect was also present but less marked in ONTARGET, which studied a population with less advanced renal disease and <40% prevalence of diabetes.

#### MR antagonists

Due to the pivotal role of aldosterone in the control of potassium homoeostasis, MR antagonist (MRA) therapy is a risk factor for hyperkalemia. In a meta-analysis of RCTs conducted in >16 000 patients with heart failure or after myocardial infarction, hyperkalemia (defined by the individual studies as [K^+^] >5.5 or 6.0 mM) occurred in 4.3% of patients allocated to placebo *versus* 9.3% of patients allocated to MRAs [48].

Famously, dangerous hyperkalemia became more common after the publication of the Randomized Aldactone Evaluation Study (RALES) study, which demonstrated a morbidity and mortality benefit from spironolactone therapy in heart failure with reduced ejection fraction (HFrEF). Large-scale data linkage in a Canadian population revealed that the rates of both hospitalization for hyperkalemia and in-hospital hyperkalemia-related mortality more than doubled after the publication of RALES in September 1999 [49].

In animal models and in Phase 1/2 clinical trials, novel non-steroidal MR antagonists have a more favourable therapeutic index than spironolactone or eplerenone—that is, they carry a lower risk of hyperkalemia at therapeutic doses in heart failure and proteinuric CKD [50, 51].

#### Other drugs

Large data-linkage studies are helping to quantify the risk of hyperkalemia associated with other medications. For example, trimethoprim prescription for urinary tract infection (compared with alternative antibiotics) was associated with a small increase in the odds of hyperkalemia in patients >65 years of age. For every 1000 infections, treatment with trimethoprim rather than amoxicillin results in one to two cases of hyperkalemia in an unselected population. In keeping with the pervasive concept that risk is potentiated when drugs that predispose to hyperkalemia are prescribed in combination in patients also taking RAS inhibitors or MRAs, trimethoprim would cause ∼18 additional cases of hyperkalemia [52].

### Other risk factors

Diabetes mellitus is a risk factor for hyperkalemia (independent of RAS blockade) because of its association with hyporeninemic hypoaldosteronism. This state is presumed to arise from decreased sympathetic drive to renin secretion (in diabetic autonomic neuropathy), decreased capacity to synthesise renin because of injury to the juxtaglomerular apparatus (in afferent arteriolar hyalinosis and diabetic nephropathy) and a decreased volume stimulus to renin release because of chronic renal salt retention [53, 54].

Hyperkalemia may also arise, via some unknown mechanism, in the ‘hungry bones’ syndrome after parathyroidectomy for secondary hyperparathyroidism. The risk may be particularly high in patients who have been treated with cinacalcet [55].

### Risk factors for hyperkalemia in dialysis patients

Patients receiving dialysis for end-stage renal failure form a group worthy of special consideration, because the risk of hyperkalemia can be influenced by variables in dialysis therapy. For example, in patients receiving maintenance hemodialysis, serum [K^+^] is influenced by time since the last dialysis therapy, being higher after a 3-day than a 2-day gap (reviewed in Rhee [56]). Interestingly however, the potassium content of the hemodialysis fluid does not appear to be a major determinant of serum [K^+^], as measured 2 or 3 days later before the next dialysis session [57].

## CONSEQUENCES OF HYPERKALEMIA

The most notorious consequence of hyperkalemia is that of potentially fatal cardiac dysrhythmia. However, there are several other consequences worthy of discussion. Hyperkalemia is associated with increased mortality (although we do not know whether hyperkalemia *causes* increased mortality outside of the context of cardiac arrhythmia in extreme hyperkalemia). Consequently, hyperkalemia—or the fear of hyperkalemia—may prompt changes in prescribing practice (e.g. avoiding RAS blockade and MRAs). Finally, recent data show that hyperkalemia can cause renal tubular acidosis and may contribute to peripheral neuropathy in CKD patients.

### Mortality: epidemiology

Large observational studies demonstrate an association between hyperkalemia and an increased risk of death [39, 58, 59]. In a meta-analysis of data from >1.2 million individuals with CKD, serum [K^+^] exhibited a U-shaped relationship with all-cause and cardiovascular mortality, with a nadir at ∼4.2 mM. The adjusted hazard ratio for all-cause mortality was ∼1.22 for [K^+^] >5.5 mM [39]. This association persisted after adjustment for a large number of covariates [39, 60]. However, as there have been no large interventional studies testing the effects of potassium-lowering therapies (or of plasma [K^+^] targets) *per se* on mortality, we do not know whether this association is due to residual confounding (i.e. whether or not mild-to-moderate hyperkalemia causes an increased risk of death).

The most plausible mechanism whereby hyperkalemia could cause death is by the induction of fatal cardiac arrhythmia. In patients with acute myocardial infarction, hyperkalemia did indeed increase the risk of ventricular arrhythmias [58]. However, there are some observations to suggest that any mortality risk associated with hyperkalemia cannot be solely attributed to arrhythmogenic death. For example, in a racially diverse population of community-dwelling older adults, hyperkalemia was not associated with sudden cardiac death, whereas it was associated with death from cancer and other non-cardiac causes [60]. Moreover, adverse outcomes have been associated with higher plasma [K^+^] values that remain well within the reference range [39, 60, 61]. Therefore we still have much to learn about the methodology of epidemiological studies in this area and/or the pathogenic consequences of hyperkalemia [62].

### Cardiac dysrhythmia: mechanism

**FIGURE 2:**
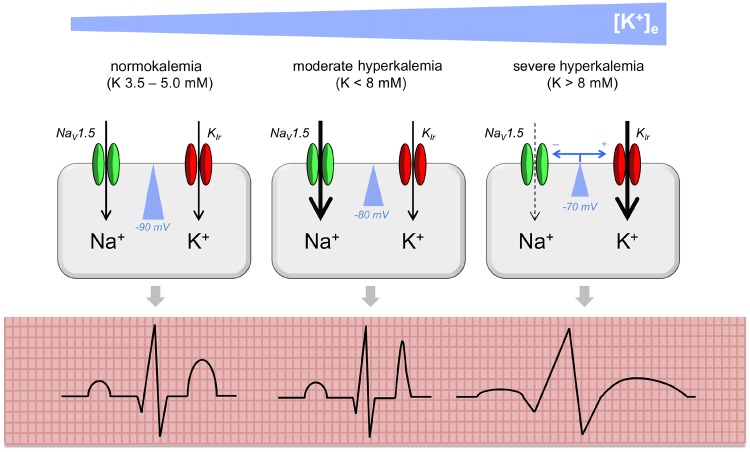
Mechanism of cardiac arrhythmia in hyperkalemia. In normokalemia, the cell membrane of the cardiomyocyte is polarized (resting potential around −90 mV). In moderate hyperkalemia, the cell membrane becomes partially depolarized, bringing the resting potential closer to the threshold potential for AP initiation. Therefore fast sodium channels (Na_v_1.5) are activated more readily, increasing excitability and conduction velocity. This manifests as T wave peaking on the ECG as a mass of ventricular cardiomyocytes undergo (synchronous) early repolarization. In severe hyperkalemia, voltage-dependent inactivation of Na_v_1.5 channels and activation of inwardly rectifying potassium channels (K_ir_) lead to reductions in conduction velocity and can render cells refractory to excitation. This manifests as broadening of ECG complexes and/or conduction blocks. This figure represents an idealized model, as there is poor correlation between ECG features and the degree of hyperkalemia.

An increase in [K^+^]_e_ has several consequences for the myocardial action potential (AP; Figure 2). These are driven by depolarization of the resting (RP) and activation of inward rectifier potassium channels (carrying currents *I*_K1_ and *I*_Kr_).

Depolarization of the RP exerts a biphasic effect on myocardial excitability and conduction velocity [63–65]. Modest increases in [K^+^]_e_ (up to ∼8 mM) increase excitability and conduction velocity as the RP moves closer to the threshold potential for AP initiation [via activation of voltage-gated Na^+^ channels (Na_V_1.5)]. Further increases in [K^+^]_e_ reduce conduction velocity or even prevent AP initiation (at [K^+^]_e_ ∼14 mM), due to the steady-state inactivation of Na_V_1.5 channels at depolarized membrane potentials [63, 66]. Activation of inward rectifier K^+^ currents during repolarization (AP Phases 3 and 4) induces rapid repolarization and retards diastolic depolarization of Purkinje fibres.

The net effect is that as [K^+^]_e_ rises modestly, the AP duration shortens, manifesting as peaked T-waves on an electrocardiogram (ECG), as a mass of myocardium undergoes premature synchronous repolarization [64, 65]. Furthermore, conduction velocity increases so that cardiac conduction blocks may resolve (hence why cardiologists request measures to increase plasma [K^+^] to high–normal in bradyarrhythmias). These pro-excitatory changes may induce fibrillation or tachyarrhythmias.

As [K^+^]_e_ increases still further, conduction velocity decreases (broadening the P and QRS complexes) and the refractory period increases, promoting cardiac conduction blocks. As depolarization merges with (premature) repolarization, there may be Q–T shortening (and apparent ST segment elevation) or the classic ‘sine wave’ ECG [64].

Thus hyperkalemia may manifest as any of various ECG abnormalities: P, QRS, ST or T wave changes, resolution of conduction blocks, new conduction blocks, asystole, atrial fibrillation, ventricular fibrillation or ventricular tachycardia. However, the sensitivity and specificity of ECG changes for hyperkalemia (and for cardiac death in hyperkalemia) are poor, and there are many case reports of normal or near-normal ECGs in patients with severe hyperkalemia [65, 67].

The pro-arrhythmogenic effects of hyperkalemia may be reversed by therapy with calcium (typically administered as chloride or gluconate salts) or sodium (typically as sodium bicarbonate) [68, 69].

### Peripheral neuropathy

The effects of hyperkalemia on myocardial excitability can be dramatic, but there is emerging evidence that hyperkalemia may also exert clinically important effects on neuronal excitability. Median nerve conduction studies in hemodialysis patients have demonstrated pre-dialysis axonal depolarization (when serum [K^+^] was ∼5.4 mM) [70]. Using an elegant protocol in which the nerve conduction studies were repeated after dialysis against a high-dialysate [K^+^] (effectively a ‘potassium clamp’) and again after dialysis against a low-dialysate [K^+^], Arnold *et al.* [71] demonstrated that a normal electrophysiological profile could be restored by lowering serum [K^+^] but not by the clearance of other uremic toxins. They speculate that chronic hyperkalemia could contribute to the pathogenesis of uremic neuropathy and myopathy. In support of this, dietary potassium restriction improved peripheral nerve function in patients with CKD in a single-blind RCT [71].

### Renal tubular acidosis

The combination of hyperkalemia and impaired renal acid excretion is termed type IV renal tubular acidosis. In 1970s–1990s, experiments in cultured cells, intact rodents, dogs and humans demonstrated that hyperkalemia can impair ammoniagenesis in the proximal renal tubule [72–74]. Because net renal acid excretion is the sum of ammonium and titratable acid excretion minus bicarbonate excretion, this suggests that hyperkalemia *per se* could cause renal tubular acidosis. However, with otherwise normal renal and adrenal function, acid–base homoeostasis is maintained in hyperkalemia by compensatory mechanisms (largely driven by increased aldosterone production). Consequently, type IV RTA is invariably encountered clinically in the context of hypoaldosteronism, either true or functional [74].

Compelling evidence that hyperkalemia can cause renal tubular acidosis was recently obtained from a mouse model of PHAII [75]. These mice carry a mutation that drives constitutive activation of the thiazide-sensitive co-transporter, NCC. They exhibited hyperkalemia, hyperchloremic metabolic acidosis and reduced urinary ammonia excretion, phenomena that were all corrected by dietary potassium restriction or thiazide administration. Furthermore, this model was exploited to determine the molecular mechanisms whereby hyperkalemia impairs renal ammonium excretion. Mice had reduced expression of ammoniagenic enzymes (phosphoenolpyruvate carboxykinase and phosphate-dependent glutaminase) and increased expression of an ammonia-recycling enzyme (glutamine synthetase) in the proximal tubule, changes that would be expected to result in reduced ammonia production. Expression of transport proteins in the collecting duct (Rhcg and H^+^-ATPase) was altered in a manner expected to reduce ammonia secretion into the urine. These molecular changes were also corrected when plasma [K^+^] was normalized with thiazide treatment.

### Consequences of hyperkalemia in patients receiving dialysis

Again, we can consider patients on dialysis as a special group for two reasons: first, the prevalence of hyperkalemia (pre-dialysis serum [K+] >5.5 mM in ∼20% of hemodialysis patients) and second, the prevalence of comorbidities that might potentiate any risk of cardiac arrhythmia and death (e.g. left ventricular hypertrophy) [57, 76].

In patients receiving maintenance hemo- and peritoneal dialysis, hyperkalemia has been associated with an increased risk of all-cause mortality, cardiac arrhythmia and hospitalization [56, 57, 76]. In hemodialysis, the risks of hyperkalemia may be augmented during the long interdialytic gap. In an observational study of >50 000 hemodialysis patients, hyperkalemia (>5.5 mM pre-dialysis) was associated with an increased risk of hospitalization in the subsequent 4 days and this risk was highest for [K^+^] measured on a Friday (i.e. before a 3-day gap) than for [K^+^] measured on a Monday or Wednesday (i.e. before a 2-day gap) [76].

### Changes in prescribing practice

#### Prescribing behaviour in hyperkalemia

The risk of hyperkalemia may deter physicians or patients from choosing certain medications. For example, only 40% of eligible patients with heart failure were prescribed an MRA in a Swedish registry of >11 000 heart failure patients. Creatinine clearance <60 mL/min was associated with non-prescription of MRA therapy (but serum [K^+^] was not) [77]. A systematic review of observational and registry data from >80 000 patients with HFrEF estimated that the ‘treatment gap’ (i.e. proportion of eligible patients who were not prescribed treatments for HFrEF) was 13.1% for ACEis/ARBs and 16.8% for MRAs and that renal failure was associated with non-prescription [78].

In an observational study of >190 000 UK patients with pre-dialysis CKD Stages 3–5, serum [K^+^] exhibited a J-shaped relationship with rates of RASi discontinuation [79]. The adjusted incidence rate ratios for RASi discontinuation if [K^+^] within 30 days >6.0 mM was ∼4.4 (compared with the reference category 4.5–5.0 mM). This value was ∼1.9 for [K^+^] 5.5–6.0 mM. Other significant associations with RASi discontinuation were lower GFR, diabetes mellitus, use of diuretics and male sex.

#### Strategies to avoid ‘deprescribing’ in hyperkalemia

RASi and MR antagonists might be legitimately withheld due to well-justified fears of hyperkalemia in patients with significant risk factors or in patients who prioritize avoidance of hyperkalemia (or other factors such as tablet burden) over any potential prognostic benefit. On the other hand, they may be withheld because of an exaggerated fear of hyperkalemia on the part of the physician or patient. To our knowledge, there have been no robust studies that have succeeded in exploring the underlying reasons for this gap in a large population.

Several potential strategies could be used to minimize ‘deprescribing’ and thus realize the potential benefit of RASis and MRAs in target patient groups. First, we could ensure that prescribing decisions are made in the context of accurate data regarding the presence—or risk—of hyperkalemia in any individual patient. Large datasets have been used to construct hyperkalemia ‘risk scores’ [44, 79]. Emerging technologies (such as wearable and needle-free potassium sensors) providing real-time [K^+^]_e_ data could allow RASis/MRAs to be omitted only on hyperkalemic days rather than being permanently discontinued in high-risk patients.

Second, novel alternative drugs, such as non-steroidal MR antagonists, may confer less risk of hyperkalemia than conventional agents. These are being tested in clinical trials.

Third, one should not forget tried-and-trusted methods for lowering potassium levels. Effective dietary advice and dietetic input can play a vital role in limiting hyperkalemia. Administration of loop and thiazide diuretics can be a useful strategy in selected patients.

Finally, hyperkalemia could be avoided by the co-prescription of tolerable potassium binders (patiromer or sodium zirconium cyclosilicate). This strategy was successful in preventing RASi deprescribing in a small RCT [80]. So far, these novel agents appear to be a valuable addition to our therapeutic armoury. However, we should be relatively cautious about the introduction into widespread clinical practice and remain vigilant for side effects that may emerge during post-marketing surveillance. In clinical trials, patiromer was associated with adverse reactions including hypomagnesemia and gastrointestinal upset and sodium zirconium cyclosilicate was associated with hypokalemia and oedema [81, 82].

## CONCLUSIONS

In classical models of potassium homoeostasis, aldosterone exerts negative feedback control of renal potassium excretion. There is an increasing appreciation that feedforward gut-to-kidney signalling plays an important role.

Hyperkalemia occurs when renal potassium excretion is limited by reductions in GFR, tubular flow, distal sodium delivery or the expression of (aldosterone-sensitive) ion transporters in the distal nephron. Accordingly, the major risk factors for hyperkalemia are renal failure, diabetes mellitus, adrenal disease and the use of ACEis, ARBs or potassium-sparing diuretics.

Hyperkalemia is associated with an increased risk of death and this is explicable only in part by hyperkalemia-induced cardiac arrhythmia. In addition to its well-established effects on cardiac excitability, hyperkalemia may also contribute to peripheral neuropathy and cause renal tubular acidosis. Fear of hyperkalemia might also contribute to the underprescription of potentially beneficial medications, particularly in HFrEF. The novel potassium binders may find a role in maximizing RASi and MRA use in heart failure and proteinuric kidney disease. We summarize the points that are most relevant for clinical practice in Table 2.

**Table 2:** Clinical ‘pearls’

Oral potassium loads induce a lesser increase in plasma [K^+^] when administered along with a complex meal (in healthy volunteers) Intravenous 0.9% NaCl is more likely than plasmalyte to cause an acute increase in plasma [K^+^] Renal potassium excretion can be improved by measures that increase tubular flow rate, distal tubular sodium delivery and urinary alkalinization CNIs can cause hyperkalemia by activating the thiazide-sensitive NaCl co-transporter and NCC In CKD, the relative risk of hyperkalemia approximately doubles for every decrease in eGFR of 15 mL/min The risk of developing hyperkalemia after initiation of RAS blockade can be predicted with a score derived from six risk factors: male sex, baseline [K^+^], eGFR, diabetes, heart failure and use of potassium-sparing diuretics The risk of hyperkalemia with dual RAS blockade (as opposed to single-agent blockade) is approximately doubled in RCTs in diabetic nephropathy The sensitivity and specificity of ECG changes for hyperkalemia are poor In patients on maintenance hemodialysis, the risk of hospitalization associated with hyperkalemia is higher when the hyperkalemia is detected prior to the long (3 days) interdialytic gap

CNIs, calcineurin inhibitors.
